# The dynamic cellular and molecular features during the development of radiation proctitis revealed by transcriptomic profiling in mice

**DOI:** 10.1186/s12864-022-08668-5

**Published:** 2022-06-09

**Authors:** Qingzhi Zeng, Jingyang Cheng, Haiyong Wu, Wenfeng Liang, Yanmei Cui

**Affiliations:** Guangdong Provincial Key Laboratory of Colorectal and Pelvic Floor Diseases, Guangdong Institute of Gastroenterology, The Sixth Affiliated Hospital, Sun Yat-sen University, Guangzhou, 510655 China

**Keywords:** Radiation proctitis, RNA-seq, Molecular feature, Mouse model

## Abstract

**Background:**

Radiation proctitis (RP) is the most common complication of radiotherapy for pelvic tumor. Currently there is a lack of effective clinical treatment and its underlying mechanism is poorly understood. In this study, we aimed to dynamically reveal the mechanism of RP progression from the perspective of RNomics using a mouse model, so as to help develop reasonable therapeutic strategies for RP.

**Results:**

Mice were delivered a single dose of 25 Gy rectal irradiation, and the rectal tissues were removed at 4 h, 1 day, 3 days, 2 weeks and 8 weeks post-irradiation (PI) for both histopathological assessment and RNA-seq analysis. According to the histopathological characteristics, we divided the development process of our RP animal model into three stages: acute (4 h, 1 day and 3 days PI), subacute (2 weeks PI) and chronic (8 weeks PI), which could recapitulate the features of different stages of human RP. Bioinformatics analysis of the RNA-seq data showed that in the acute injury period after radiation, the altered genes were mainly enriched in DNA damage response, p53 signaling pathway and metabolic changes; while in the subacute and chronic stages of tissue reconstruction, genes involved in the biological processes of vessel development, extracellular matrix organization, inflammatory and immune responses were dysregulated. We further identified the hub genes in the most significant biological process at each time point using protein-protein interaction analysis and verified the differential expression of these genes by quantitative real-time-PCR analysis.

**Conclusions:**

Our study reveals the molecular events sequentially occurred during the course of RP development and might provide molecular basis for designing drugs targeting different stages of RP development.

**Supplementary Information:**

The online version contains supplementary material available at 10.1186/s12864-022-08668-5.

## Background

Radiotherapy is one of the main treatments for pelvic malignant tumors [1]. However, while radiotherapy is used to kill tumor cells, it will also inevitably cause damage to the adjacent normal tissues, leading to the occurrence of radiation-induced complications [2]. Radiation proctitis (RP) is the rectal injury caused by pelvic radiotherapy, which can be generally classified as acute radiation proctitis (ARP) and chronic radiation proctitis (CRP) according to the onset time and related clinical symptoms [3]. The ARP may appear within hours to days after radiation exposure and the clinical manifestations include abdominal pain, diarrhea and tenesmus. Most of these symptoms may resolve within 3 months. However, some patients will develop into CRP, which usually appears more than 6 months after the end of radiotherapy, and can manifest by even serious complications such as rectal stricture, intestinal perforation and bleeding, which seriously affect the quality of life of patients [3–5].

The pathological features of ARP include: mucosal surface erosion, submucosal edema, and inflammatory cell infiltration in the lamina propria; while CRP is characterized by mucosal atrophy, irregularly arranged crypts, vascular stenosis and occlusion, and interstitial fibrosis [3, 6].

Although the clinical features and pathological changes of RP are well documented, the molecular mechanism underlying the progression of RP is still poorly understood. There have studies focusing on uncovering the factors that play a regulatory role either in fibrosis or in intestinal epithelial regeneration after radiation injury [7–11]. However, these studies look at a single event in either acute or chronic stage, and few studies have been designed to dynamically characterize the molecular events that occur during the progression of RP. Here, we use an RP animal model to dynamically analyze the transcriptome gene expression profiles at different stages of disease development by setting multiple time points after irradiation, aiming to provide insight into the molecular basis of RP development and ultimately help to explore potential therapeutic targets.

## Results

### The pathological features of rectal tissue at different time points post-irradiation (PI) in the mouse model recapitulate the features of human ARP and CRP

**Fig. 1:**
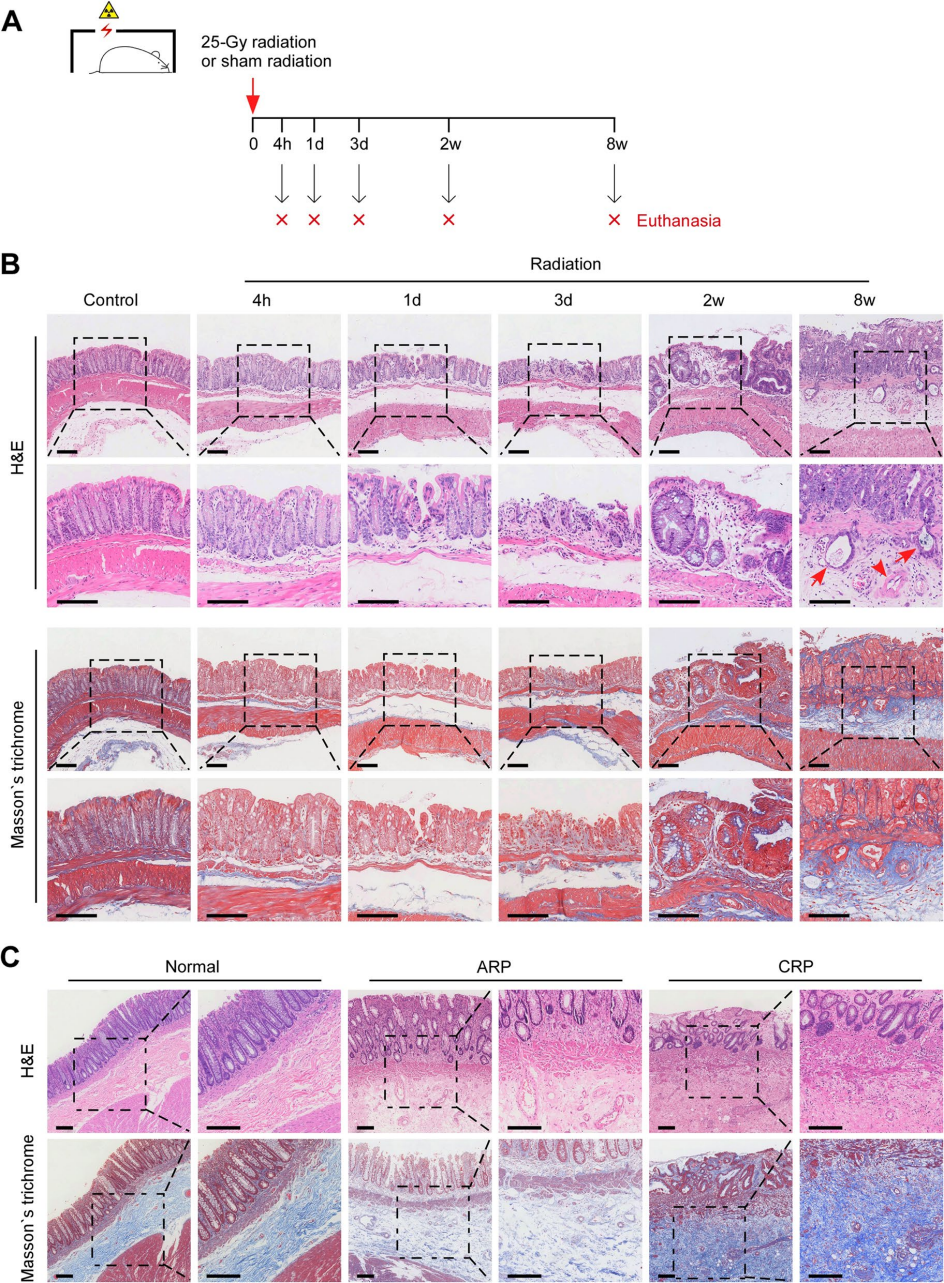
The pathological features of RP mouse model. **A** Experimental design of the animal model. **B** Representative images of H&E and Masson’s trichrome staining of the rectal tissues in control (4 h) and radiation groups at each indicated time point. Scale bars, 100 μm. **C** Representative images of H&E and Masson’s trichrome staining of ARP, CRP lesions and normal colon tissue of human samples. Scale bars, 100 μm. The red arrows in **B** indicate mucosal glands inserted into the submucosa, and the red arrow head in **B** indicates vascular lesion

In order to investigate the intrinsic pathogenesis of RP in its early and late stages, we employed a RP mouse model reported in previous study [7] and examined the injury rectal tissue at different time points (4 h, 1 day, 3 days, 2 weeks and 8 weeks) after a single dose of 25 Gy rectal irradiation. Age-matched sham-irradiated mice for each time point were used as control group (Fig. 1 A). Histological staining revealed that the irradiated rectal tissues showed slight mucosal edema and epithelial disruption at 4 h PI, but marked mucosal defects and edema with inflammatory cell infiltration at 1 and 3 days PI, which mimic the pathological characteristics of human colorectal tissue obtained from adjacent non-cancerous colorectal tissue of rectal cancer patient who underwent radiotherapy before surgery and subsequently suffered from ARP (Fig. 1B-C).

At 2 weeks PI, there was still edema and presented regenerated crypts with irregular size and arrangement in the mucosal layer, and Masson’s trichrome staining showed slight collagen fibers deposition in the submucosa (Fig. 1B). We defined this phase as the subacute stage, which is the transition from acute to chronic stage. While at 8 weeks PI, the hyperplastic crypts were extremely irregularly arranged, and the mucosal glands inserted into the submucosa. More characteristically, there was substantial fibrosis and significant stenosis of small blood vessels in the submucosal layer, which also recapitulate the histopathological features of tissue lesions from human CRP (Fig. 1B-C). Therefore, our mouse model well reproduces the pathological characteristics of human ARP and CRP, and could serve as a suitable model to investigate the molecular basis of RP progression.

#### Transcriptome analysis of gene expression changes in the course of RP development

**Fig. 2:**
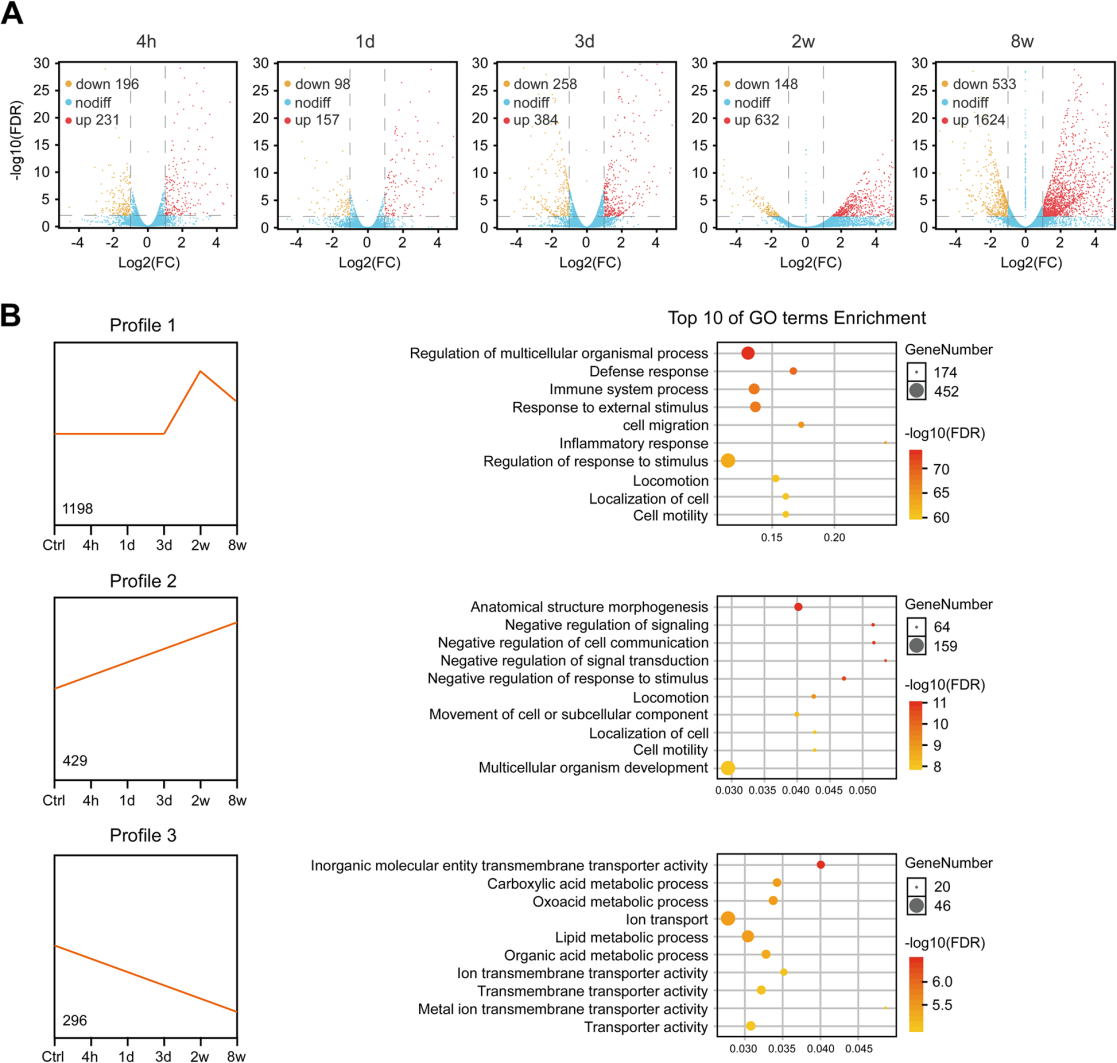
Analysis of DEGs at each time point PI. **A** Volcano plots of genes changed at each indicated time point PI. The numbers of up- or down-regulated genes (|Log2FC|>1 and FDR < 0.01) in each time point are indicated at the top left of the graphs. FC: fold change. **B** Gene profiles classified by STEM algorithm. Profiles were sorted according to the number of genes contained, and the top 3 with the largest number of genes were shown. The graphs (left) show the trends of gene expression change over time after exposure compared to sham-irradiated controls at 4 h. For each profile, the vertical axis represents the gene expression levels based on FPKM and the horizontal axis represents time points post-irradiation. The number in the lower left of the box represents the gene number contained in each profile. The bubble chart (right) shows the top10 most enrichment GO terms in each profile

We next performed RNA-seq of the freshly collected rectal tissues from both irradiated group and sham-irradiated control group at each time point. After filtering out adapters or low quality bases, a total of 19,690 genes were identified from reads counting based on the RNA-seq data analysis (Additional file 1). The numbers of differentially expressed genes (DEGs) in the irradiated groups were 427(231 up and 196 down), 255 (157 up and 98 down), 642 (384 up and 258 down), 780 (632 up and 148 down) and 2157 (1624 up and 533 down) at the time points of 4 h, 1 day, 3 days, 2 weeks and 8 weeks, respectively, compared with their respective control groups (Fig. 2 A).

In order to characterize the changing trend of these DEGs along with the progression of RP, we performed Short Time-series Expression Miner (STEM) analysis. DEGs were grouped in time-related expression profiles that were then analyzed for enrichment in Gene Ontology (GO) terms. A total of eight statistically significant gene profiles were identified (Fig. 2B and Additional file 2: Figure S1). The expression of genes in profile 1, which included 1198 genes, remained unchanged during the acute injury period from 4 h to 3 days, then suddenly increased at 2 weeks and partially recovered at 8 weeks, which were mostly enriched in the GO terms: regulation of multicellular organismal process, defense response, immune system process and response to external stimulus (Fig. 2B). Moreover, 429 genes in profile 2 showed a gradual increase throughout the course of disease progression, which were associated with anatomical structure morphogenesis and negative regulation of signaling and cell communication; whereas 296 genes in profile 3 showed a gradually down-regulated trend, which were mainly involved in inorganic molecular entity transmembrane transporter activity and metabolic process (Fig. 2B). Profile 4 containing 208 genes which were associated with cell cycle process showed a decrease at 4 h and remained low until 1 day, then returned to baseline at 3 days but slightly increased at 2 weeks; whereas profile 5 showed an opposite trend to profile 4, in which 143 genes classified as GO terms associated with intracellular organelles increased at 4 h PI and maintained a high level until 1 day, then began to decrease and reached the lowest level at 2 weeks, but returned to normal at 8 weeks (Additional file 2: Figure S1). In addition, 122 genes in profile 6 associated with cell cycle and DNA repair showed a zigzag trend, which decreased at 4 h, but increased at 3 days and then decreased below the baseline at 2 weeks (Additional file 2: Figure S1). The 108 genes in profile 7 mostly enriched in multicellular organism development also showed a zigzag pattern of changes, decreasing at 4 h, returning to normal at 1 day, slightly decreasing at 3 days and then rising above the baseline at 8 weeks (Additional file 2: Figure S1). Furthermore, 83 genes in profile 8 associated with brush border membrane showed a temporary decline at 4 h and recovered at 1 day, but then began to decline and dropped to much lower than normal at 8 weeks, indicating irreversible impairment of intestinal villi function in the chronic stage (Additional file 2: Figure S1).

**Fig. 3:**
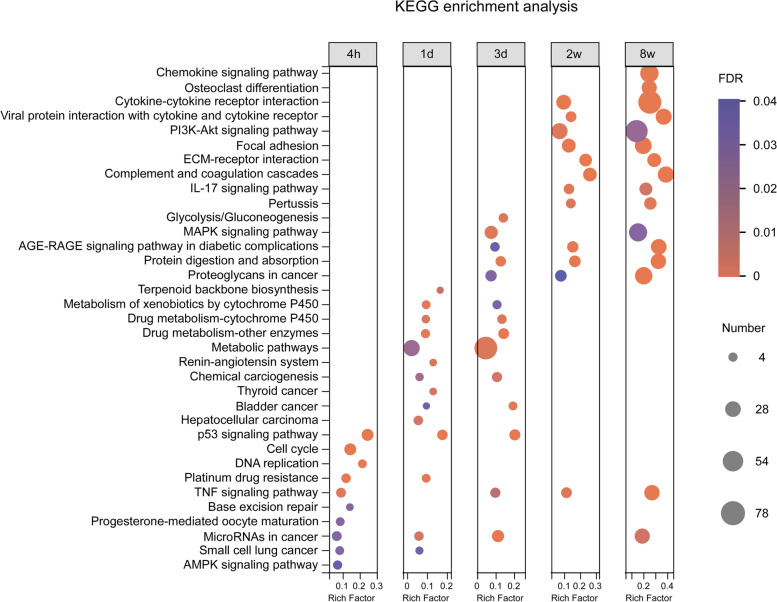
KEGG enrichment analysis of the alterations of pathways at each time point PI. The top 10 most significant enrichment KEGG pathways based on DEGs were selected for each time point, and a total of 35 different KEGG pathways are shown for all time points. The color of dots represents FDR value, and the size of dots represents the number of genes enriched in the pathway

We then conducted the Kyoto Encyclopedia of Genes and Genomes (KEGG) enrichment analysis with the DEGs at each time point to analyze the sequential activation of signaling pathways. The results showed that p53, cell cycle and TNF signaling pathways were initially dysregulated at 4 h PI; while at 1 day after exposure, terpenoid backbone biosynthesis and drug metabolism-related pathways were dominantly altered (Fig. 3). Moreover, the metabolic pathways such as Glycolysis/Gluconeogenesis and AGE-RAGE signaling pathway, as well as MAPK signaling pathway were significantly dysregulated at 3 days PI (Fig. 3). In contrast, when at 2 and 8 weeks after exposure, the activated signaling pathways were strikingly different from those in the acute phase. The most significant activated pathways at 2 weeks PI were cytokine-cytokine receptor interaction, PI3K-Akt signaling pathway, focal adhesion, ECM-receptor interaction as well as immune-related signaling pathways, which were further augmented at 8 weeks PI (Fig. 3). Thus, the dominant signaling pathways are activated sequentially as the disease progresses from acute to chronic stages.

## GO analysis of DEGs and identification of hub genes at each time point PI

**Fig. 4:**
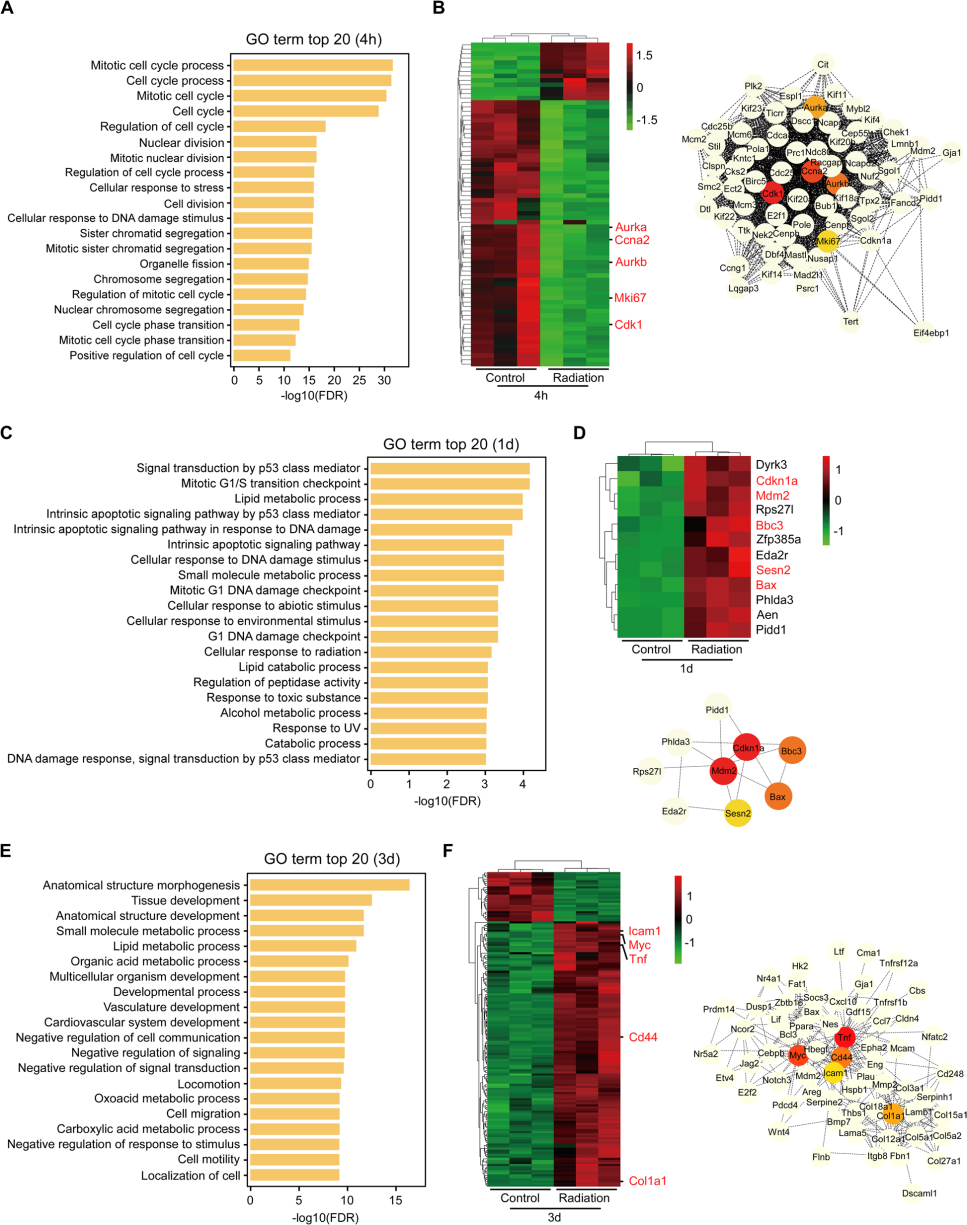
GO analysis of DEGs in the acute phase. **A** GO analysis of DEGs at 4 h PI. The top 20 GO terms with the most significant enrichment are shown. **B** Heatmap (left) and PPI analysis (right) of the genes enriched in the top 1 GO term in A. Hub genes are shown in color. **C** GO analysis of DEGs at 1 day PI. The top 20 GO terms with the most significant enrichment are shown. **D** Heatmap (upper) and PPI analysis (lower) of the genes enriched in the top 1 GO term in C. Hub genes are shown in color. **E** GO analysis of DEGs at 3 days PI. The top 20 GO terms with the most significant enrichment are shown. **F** Heatmap (left) and PPI analysis (right) of the genes enriched in the top 1 GO term in E. Hub genes are shown in color

**Fig. 5:**
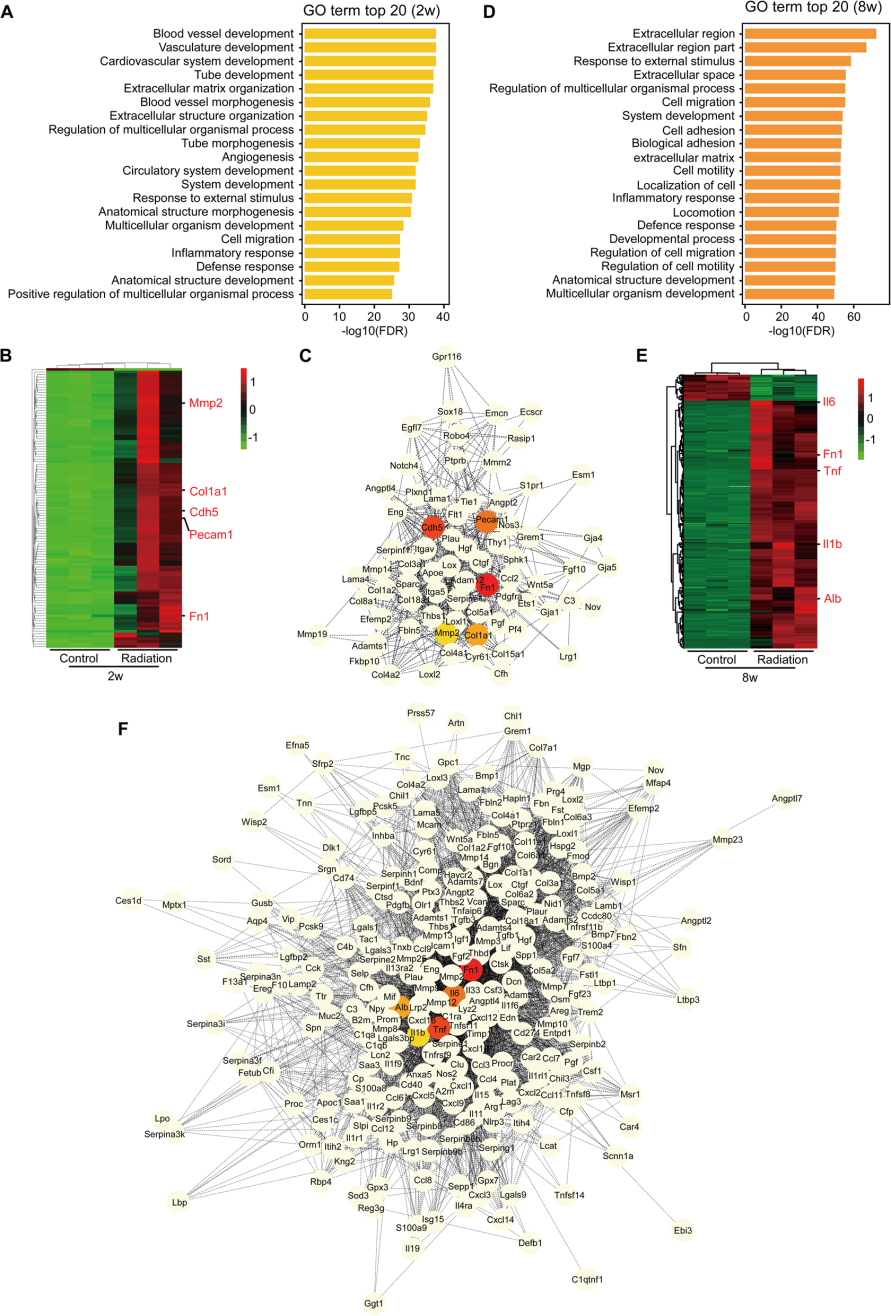
GO analysis of DEGs in the subacute and chronic phases. **A** GO analysis of DEGs at 2 weeks PI. The top 20 GO terms with the most significant enrichment are shown. **B**-**C** Heatmap (**B**) and PPI analysis (**C**) of the genes enriched in the top 1 GO term in A. Hub genes are shown in color. **D** GO analysis of DEGs at 8 weeks PI. The top 20 GO terms with the most significant enrichment are shown. **E**-**F** Heatmap (**E**) and PPI analysis (**F**) of the genes enriched in the top 1 GO term in D. Hub genes are shown in color

We further performed the GO analysis based on the DEGs at each time point PI. The top 20 most significantly enriched GO terms at each time point were shown (Fig. 4). At 4 h PI, the altered genes in the irradiation group were mainly enriched in the biological processes of cell cycle and DNA damage response (Fig. 4 A). We next showed the alteration genes in mitotic cell cycle process which was the top 1 enrichment GO term and also did protein-protein interaction (PPI) analysis of these genes (Fig. 4B and Additional file 2: Table S1). The result showed that Cdk1, Ccna2, Aurkb, Aurka and Mki67 that were down-regulated in the irradiated group were located at central hubs and might play critical roles in cell cycle process in response to the radiation injury (Fig. 4B). At 1 day PI, the altered genes were mainly involved in signal transduction by p53 class mediator, mitotic G1/S transition checkpoint and lipid metabolic process, and Mdm2, Cdkn1a, Bbc3, Bax and Sesn2 that were up-regulated in the irradiated group were further identified as hub genes in the p53-mediated signal transduction (Fig. 4 C-D and Additional file 2: Table S1). While at 3 days PI, the alteration genes in the radiation group were mainly enriched in the anatomical structure morphogenesis, tissue development as well as metabolic processes such as small molecule and lipid metabolisms, and the hub genes in the anatomical structure morphogenesis were Tnf, Myc, Cd44, Col1a1 and Icam1 which were all up-regulated in the irradiated group (Fig. 4E-F and Additional file 2: Table S1). Interestingly, at 2 weeks PI, the altered genes were mostly enriched in the biological processes related to vessel development, and Fn1, Cdh5, Pecam1, Col1a1 and Mmp2 that were all up-regulated in the irradiated group were identified as hub genes in the blood vessel development process (Fig. 5 A-C and Additional file 2: Table S1). Furthermore, at 8 weeks PI, the most significant changes in the irradiated group were in the extracellular region and the biological process in response to external stimulus. PPI analysis indicated that the genes Fn1, Il6, Tnf, Alb and Il1b that were over-expressed in the irradiated group were located at central hubs and might play key regulatory roles in the GO term “extracellular region” (Fig. 5D-F and Additional file 2: Table S1).

**Fig. 6:**
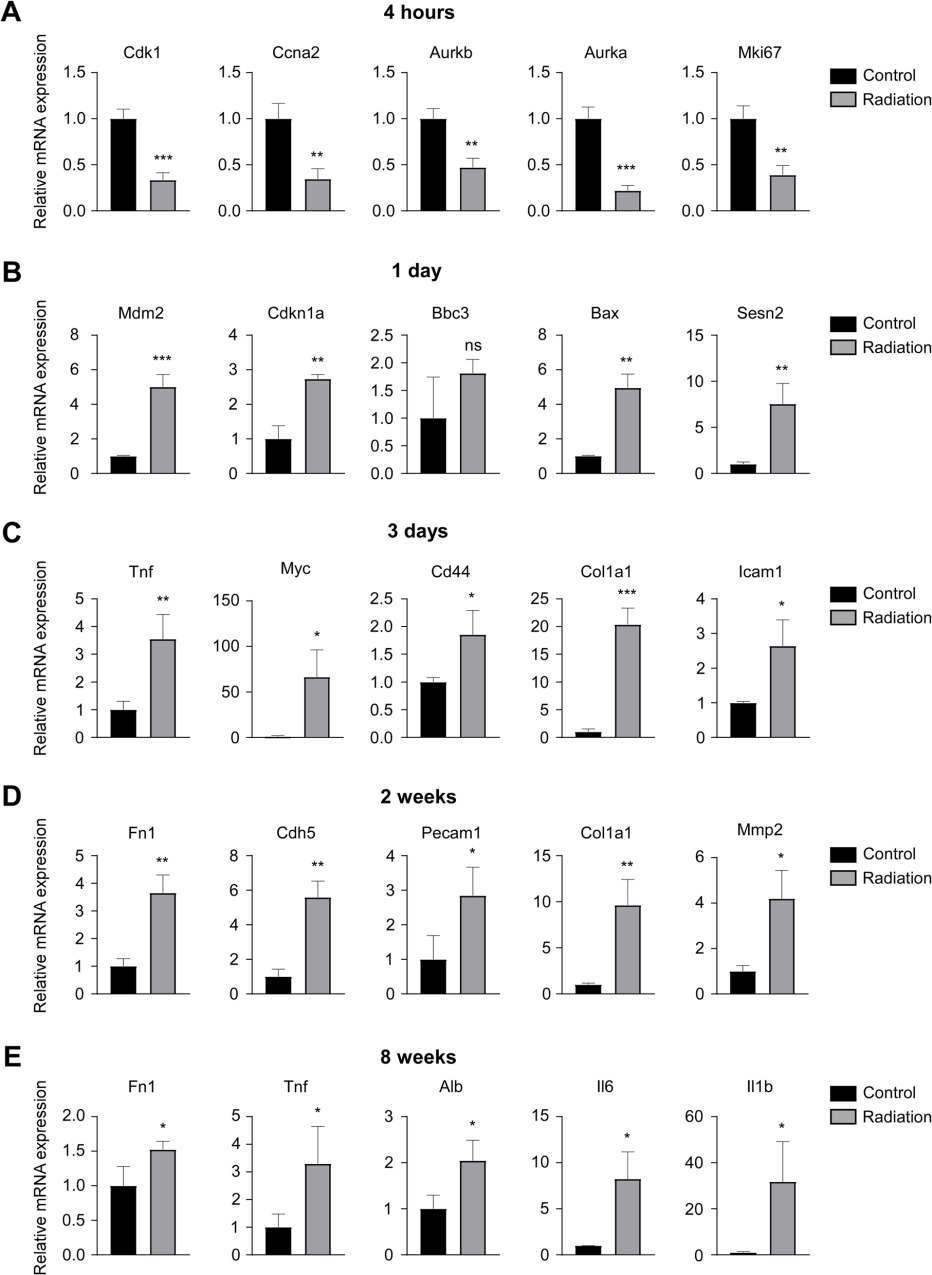
Verification of the alteration of the hub genes by qRT-PCR analysis. **A** The mRNA expression levels of Cdk1, Ccna2, Aurkb, Aurka and Mki67 in the rectal tissues of mice in control and radiation groups at 4 h PI. **B** The mRNA expression levels of Mdm2, Cdkn1a, Bbc3, Bax and Sesn2 in the rectal tissues of mice in control and radiation groups at 1 day PI. **C** The mRNA expression levels of Tnf, Myc, Cd44, Col1a1 and Icam1 in the rectal tissues of mice in control and radiation groups at 3 days PI. **D** The mRNA expression levels of Fn1, Cdh5, Pecam1, Col1a1 and Mmp2 in the rectal tissues of mice in control and radiation groups at 2 weeks PI. **E** The mRNA expression levels of Fn1, Tnf, Alb, Il6 and Il1b in the rectal tissues of mice in control and radiation groups at 8 weeks PI. *n* = 3 for each time points. Data are shown as means ± SD. Statistical analyses were performed by unpaired Student’s t-test. ns, not significant. *p < 0.05, **p < 0.01, ***p < 0.001

We next verified these hub genes identified at each time point PI using quantitative real-time-PCR (qRT-PCR) analysis. The results showed that the cell cycle-related hub genes including Cdk1, Ccna2, Aurkb, Aurka and Mki67 were all significantly down-regulated in the irradiated group compared to control group at 4 h PI (Fig. 6 A), while hub genes involved in p53-mediated signal transduction including Mdm2, Cdkn1a, Bax and Sesn2 were all significantly up-regulated in the irradiation group at 1 day PI, except for the Bbc3 gene, which was up-regulated but not statistically significant (Fig. 6B). Moreover, the hub genes Tnf, Myc, Cd44, Col1a1 and Icam1 at 3 days PI, hub genes Fn1, Cdh5, Pecam1, Col1a1 and Mmp2 at 2 weeks PI, and hub genes Fn1, Tnf, Alb, Il6 and Il1b at 8 weeks PI were all significantly up-regulated in the irradiation groups compared to their respective control groups (Fig. 6 C-E).

### P53 is specifically activated in the acute phase of mouse RP

**Fig. 7:**
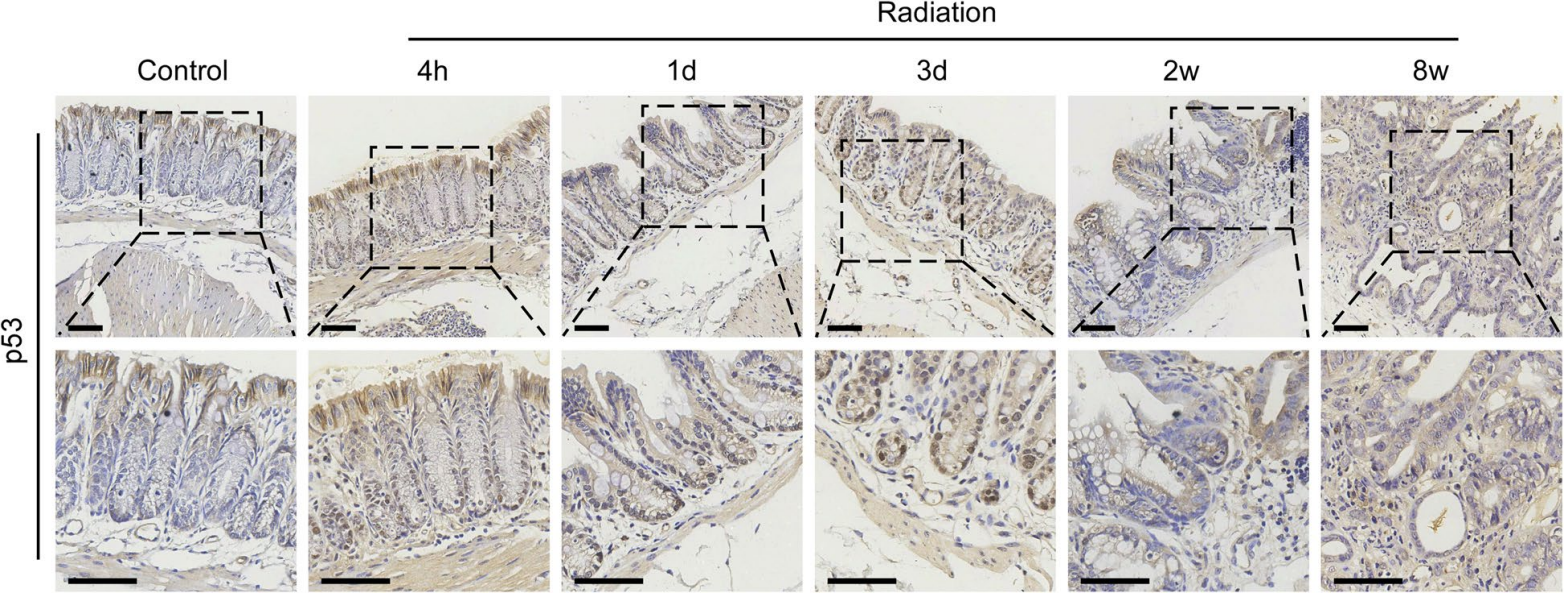
P53 is specifically activated in the acute phase in mouse RP. IHC staining was performed in the rectal tissues of mice at control and irradiated groups using anti-p53 antibody. Representative images of p53 staining of the tissue sections from control (4 h) and irradiated mice at the indicated time points are shown. Scale bars, 50 μm

We next performed immunohistochemistry (IHC) staining to detect p53 expression in the rectal tissues of mice at each time point PI. As shown in Fig. 7, we found that in the normal control, there was almost no p53 expression in the lower half of the crypt, and only moderate cytoplasmic expression of p53 at the top of the epithelium; while at 4 h, 1 day and 3 days after irradiation, a large number of nuclear p53-positive cells appeared in the lower half of the crypt, but few nuclear p53-positive cells were observed at 2 and 8 weeks after irradiation, indicating that p53 signaling pathway is specifically activated in the acute phase, which is also consistent with the KEGG analysis of our sequencing data.

## Discussion

Radiotherapy is one of the most effective means of treating pelvic malignancies [12]; however, more than 75% of patients receiving pelvic radiotherapy develop ARP, and up to 20% of the patients progress to CRP [5, 13]. The occurrence of RP seriously affects the quality of life of patients [14, 15]. However, the current clinical treatment is symptomatic without specific targeted drugs to prevent the development of RP, ultimately due to the lack of understanding of the molecular mechanism of its pathogenesis [16, 17]. In this study, we employ the RP animal model reported by Lu et al. [7] by administering irradiation to the pelvic region of mice which well mimics the clinical radiotherapy of pelvic tumors and set several observation time points to investigate the pathogenesis and molecular basis underlying ARP and CRP. Based on the histopathological characteristics of our animal model, we divide the observation time points into three stages of RP development: acute (4 h-3 days), subacute (2 weeks), and chronic (8 weeks), which could well simulate the natural evolution of human RP. We then performed transcriptome gene expression profile analysis and identified characteristically activated signaling pathways or cellular processes and key alteration genes in the acute, subacute and chronic phases, respectively, which might dynamically reveal the complex pathological process of RP.

When analyzing the RNA-seq data, we notice an interesting point that the number of DEGs increased gradually over time, except for a decrease at 1 day after irradiation. This suggests that the molecular and biological changes induced by radiation insult continue to intensify although the time away from radiation exposure gets longer, and that the disease is gradually progressing and deteriorating from acute to chronic. The number of DEGs is particularly high at 8 weeks, which is several times that of the acute phase, indicating that the most severe pathological and molecular disorders occur at the chronic phase, which also supports the fact that CRP is more harmful to patients in clinical practice [3]. Otherwise, the number of DEGs decreased at 1 day after exposure, which might be caused by the restoration of some genes that were elevated at 4 h in response to injury stress.

We also find that some groups of genes showed similar expression patterns during the progression of RP. The expression of genes related to immune response increased suddenly at 2 weeks and remained high at 8 weeks, suggesting that these genes might be involved in the pathogenesis of CRP such as fibrosis, which is consistent with previous reports that emphasized the important roles of immune cells as well as their secreted cytokines in the development of fibrosis in a variety of organ systems [18–20]. While another set of genes involved in metabolism regulation showed a gradual decrease from 4 h to 8 weeks, indicating that metabolic disorder might contribute to the whole process of RP development. Interestingly, Li et al. recently reported that the perturbation of lipid metabolism in intestinal epithelial tissue, especially the abnormal of glycerophospholipids metabolism which might be caused by the enteric dysbacteriosis of mice following 18-Gy abdominal radiation, might be closely related to the development of radiation enteritis [21]. Moreover, based on the KEGG enrichment analysis, our results showed characteristic activation of signaling pathways at different stages of RP development, specially, p53 and metabolism-related signaling pathways activated in the acute phase, and PI3K/Akt, cytokine/chemokine signaling and ECM-receptor signaling pathways mainly activated in the subacute and chronic phases, suggesting that targeting these specifically activated signaling pathways at different stages of disease development might achieve better therapeutic effects of RP.

When cells are exposed to exogenous insult such as ionizing radiation, p53 and its downstream signaling pathways are rapidly activated in response to DNA damage and increased oxidative stress, leading to widespread cell cycle arrest and programmed death of damaged cells [22–25]. Consistently, our results showed that multiple core genes that regulate cell-cycle progression including Cdk1, Ccna2, Aurka, Aurkb and Mki67 were significantly down-regulated at 4 h PI, whereas those genes involved in p53 signaling pathway, including Cdkn1a, Mdm2, Bbc3, Bax and Sesn2 were dramatically up-regulated at 1 day PI, indicating that widespread cell cycle arrest and apoptosis occur in the intestinal epithelial cells in response to the radiation damage. Previous studies have shown that the injured intestinal epithelium enters a regenerative phase 72 h after radiation exposure, which is characterized by high proliferation and crypt multiplication and regulated by developmental signaling pathways such as Wnt and Notch signaling [10, 26–29]. In line with this, we also identified the Wnt target genes Myc and Cd44, which play an important role in regulating intestinal stem cell activity [30–33], were significantly up-regulated at 3 days PI and might play a central role in regulating the structure morphogenesis at this phage.

Microvascular injury and abnormal angiogenesis have been well documented in the pathological changes of radiation injury in the late phase, which are considered to contribute largely to the progression of CRP [3, 34–36]. However, whether angiogenesis is excessive or inhibited in the process of RP is still controversial. There is more evidence in favor of promoting angiogenesis, which showed that multiple pro-angiogenic factors such as angiogenin, VEGF, CD31, fibroblast growth factor 1, and MMP8 were over-expressed and microvascular density was increased in the mucosal biopsies of human RP [37–39]. However, Wu et al. found that the expression of angiostatin was significantly up-regulated and microvascular density was decreased in the submucosa of human CRP lesions, suggesting a compromised angiogenesis [40]. Here in our study, we find that vascular dysplasia was predominate at 2 weeks PI, and the hub genes Cd31 (Pecam1), Mmp2, Cdh5, Col1a1 and Fn1 were significantly up-regulated in this process, suggesting the possibility of abnormal excessive angiogenesis. However, whether these alteration genes are simultaneously involved in the process of occlusive vasculopathy of RP requires further investigation.

One of the most prominent features of CRP is fibrosis, which is due to excessive deposition of ECM and leads to intestinal obstruction [3, 41]. Sustained immune response and over-expression of inflammatory factors have been reported to be closely related to the formation of fibrosis [19, 42, 43]. This is also supported by our observation that cytokine and immune signaling pathways probably triggered by overexpressed Il6, Tnf and Il1b were persistently activated and the ECM proteins such as Fn1 and Alb were over-accumulated at 2 and 8 weeks PI. Although the role of certain immune cells or cytokines in the pathogenesis of radiation-induced fibrosis has been revealed [7, 9, 44, 45], more efforts are needed to uncover the detail mechanism of fibrogenesis and explore effective preventive and therapeutic drugs for fibrosis in RP.

There have some limitations in our study. First, we reveal the pathogenesis of RP only from the transcriptome level, and future study should be designed to combine multi-omics analyses including transcriptome, proteomics, metabonomics and microbiome to comprehensively describe the pathogenesis of RP progression. Second, although we have identified characteristically activated signaling pathways and key altered genes at different stages of disease development in mouse model, these need to be further validated in human RP samples. And also the precise roles and mechanisms of these altered genes and signaling pathways in RP development as well as their therapeutic values in RP deserve further investigation.

## Conclusions

In summary, our study reveals the gene expression patterns during the development of RP using a mouse model by RNA-seq analysis, and reports distinct signaling pathways activated at different stages of disease development. In the acute injury period, the most important events are cell cycle arrest, p53-mediated apoptosis and dysregulation in metabolism-related pathways, whereas in the subacute and chronic phases, inflammatory and immune signaling pathways, ECM and focal adhesion are dominantly activated. In particular, changes in biological processes associated with vessel development are most pronounced in the subacute phase. Our study delineates the molecular events sequentially occurred during the development of RP and provides molecular basis for targeted therapy at different stages of RP development.

## Methods

### RP animal model

C57BL/6J mice (female) aged 8 to 10 weeks were obtained from Vital River Laboratory Animal Technology Co. (Beijing, China) and raised in specific pathogen–free condition with a 12 h light/dark cycle at the Sixth Affiliated Hospital, Sun Yat-sen University. The RP mouse model was established as previous reported [7]. In brief, mice were randomly divided into two groups. Mice in the irradiation group were anesthetized and irradiated with a dose of 25 Gy using a RS2000 X-ray Irradiator (Rad Source Technologies, Inc., Boca Raton, FL). The lower pelvic region (1 cm) containing the rectum was exposed and the rest of the body was shielded by lead plates. Mice in control group (sham-irradiated) were anesthetized without irradiation. Mice were euthanized at 4 h, 1 day, 3 days, 2 weeks and 8 weeks after irradiation in both the irradiated (n = 3 mice per time point) and time-matched control groups (n = 3 mice per time point). The same control samples were shared for 4 h and 1 day. The rectal tissues were removed and divided longitudinally into two parts for fresh freezing or fixation in 10% formaldehyde.

## Human specimens

The human normal control colon tissue was obtained from adjacent non-cancerous tissue of patients with colorectal cancer surgically resected without neoadjuvant chemoradiotherapy. The human ARP sample was obtained from adjacent non-cancerous colorectal tissue of rectal cancer patients who underwent radiotherapy before surgery and subsequently experienced symptoms of ARP. The human CRP specimen was obtained from surgically excised intestinal lesions of patients who suffered from CRP after pelvic radiotherapy for cervical cancer. The detailed information for these human specimens was listed in Additional file 2: Table S2.

## H&E and Masson’s trichrome staining

Human and murine samples were formalin-fixed, paraffin-embedded and sliced to a thickness of 4 μm, followed by H&E and Masson’s trichrome staining according to standard procedures.

## Immunohistochemistry (IHC) staining

IHC staining was carried out using SP-9000 SPlink Detection Kit (ZSGB-Bio, Beijing, China) following the manufacturer’s protocol. Rectal tissue sections of mice were detected with anti-p53 antibody (1:3000, 60283-2-Ig, Proteintech group).

## RNA-seq analysis

Total RNA from the rectal tissues of mice in both irradiated and control groups at 4 h, 1 day, 3 days, 2 weeks and 8 weeks PI was extracted using Trizol reagent (Invitrogen, Carlsbad, CA). Three biological replicates of the RNA sample were prepared for each time point. The same control samples were shared for 4 h and 1 day. A total of 27 RNA samples were collected and subjected to high-throughput RNA sequencing with Illumina HiSeq Xten platform by Mega genomics Co., LTD (Beijing, China). Sequenced reads containing adapters or low quality bases were filtered by fastp [46] (version 0.18.0). Paired-end clean reads were aligned to the mouse genome (mm9) using HISAT2. 2.4 software [47]. The mapped reads of each sample were assembled by StringTie v1.3.1 [48] in a reference-based approach. Gene expression levels were calculated using FPKM values (fragments per kilobase of exon per million mapped reads) using RSEM software [49]. Differential gene expression between two groups was analyzed by DESeq2 software [50]. The genes with the parameter of false discovery rate (FDR) < 0.01 and absolute fold change > 2 were considered differentially expressed genes. KEGG [51] and GO [52] enrichment analyses were performed using the OmicShare tools, a free online data analysis platform. Significantly enriched GO terms or pathways in DEGs comparing to the genome background were defined by hypergeometric test. The calculated *p*-value was gone through FDR correction, taking FDR ≤ 0.05 as a threshold. GO terms or pathways meeting this condition were defined as significantly enriched GO terms or pathways in DEGs. STEM was performed by STEM software [53] (version 1.3.13). The Maximum Unit Change in model profiles between time points was 2, and the maximum output profiles number was 50.

## Screening hub genes

The target genes were put into the Search Tool for the Retrieval of Interacting Genes/Proteins (STRING) [54] online database (http://stringdb.org/) to get a protein-protein interaction network, and then visualized in Cytoscape software [55] (version 3.9.0). Hub genes were calculated by CytoHubba using the Maximum Neighborhood Component method. The topological features for each PPI network were shown in Additional file 2: Table S1.

## qRT-PCR

Total RNA from the rectal tissues of mice was extracted with Trizol (Invitrogen, Carlsbad, CA). The cDNA was synthesized using ReverTra Ace® qPCR RT Master Mix with gDNA Remover (TOYOBO). qRT-PCR was performed using FastStart Essential DNA Green Master (Roche) with Quantstudio 7 flex RT-PCR systems (Applied Biosystems Inc., Foster City, CA). β-actin was used as the internal control. The sequences of the used primers were shown in Additional file 2: Table S3.

### Statistical analysis

The qRT-PCR data were analyzed by GraphPad Prism 8 (GraphPad, San Diego, CA, USA). Comparisons of gene expression levels were performed by unpaired Student’s t-test. Data were shown as mean ± SD. *P* < 0.05 was considered significant.

## Supplementary Information

**Additional file 1.** Excel file containing differential expression data for all genes across all comparison groups and time points.

**Additional file 2: Figure S1.** Gene profiles classified by STEM algorithm. **Table S1.** The average values of topological features of the PPI networks for each time point. **Table S2.** Information for the human specimens. **Table S3.** Primer sequences for qRT-PCR.

## Data Availability

The RNA-seq data generated during the current study are available in National Center for Biotechnology Information (NCBI) Gene Expression Omnibus (GEO) database with accession number GSE198331 (http://www.ncbi.nlm.nih.gov/geo/query/acc.cgi?acc=GSE198331).

## Abbreviations

ARP: Acute radiation proctitis
CRP: chronic radiation proctitis
DEGs: Differentially expressed genes
FC: Fold change
FDR: False discovery rate
GO: Gene Ontology
IHC: Immunohistochemistry
KEGG: Kyoto Encyclopedia of Genes and Genomes
PI: Post-irradiation
PPI: Protein-protein interaction
qRT-PCR: quantitative real-time-PCR
RP: Radiation proctitis
STEM: Short Time-series Expression Miner
