# Supplementary material for: The dynamic cellular and molecular features during the development of radiation proctitis revealed by transcriptomic profiling in mice
Source: BMC Genomics. 2022 Jun 9;23:431. doi: 10.1186/s12864-022-08668-5 (PMC9178886; doi:10.1186/s12864-022-08668-5)
Supplement: Supplementary file 2 — Additional file 2: Figure S1. Gene profiles classified by STEM algorithm. Table S1. The average values of topological features of the PPI networks for each time point. Table S2. Information for the human specimens. Table S3. Primer sequences for qRT-PCR. [file 12864_2022_8668_MOESM2_ESM.docx]

**Supplementary Figure and Tables**

**Supplementary Figure**

**
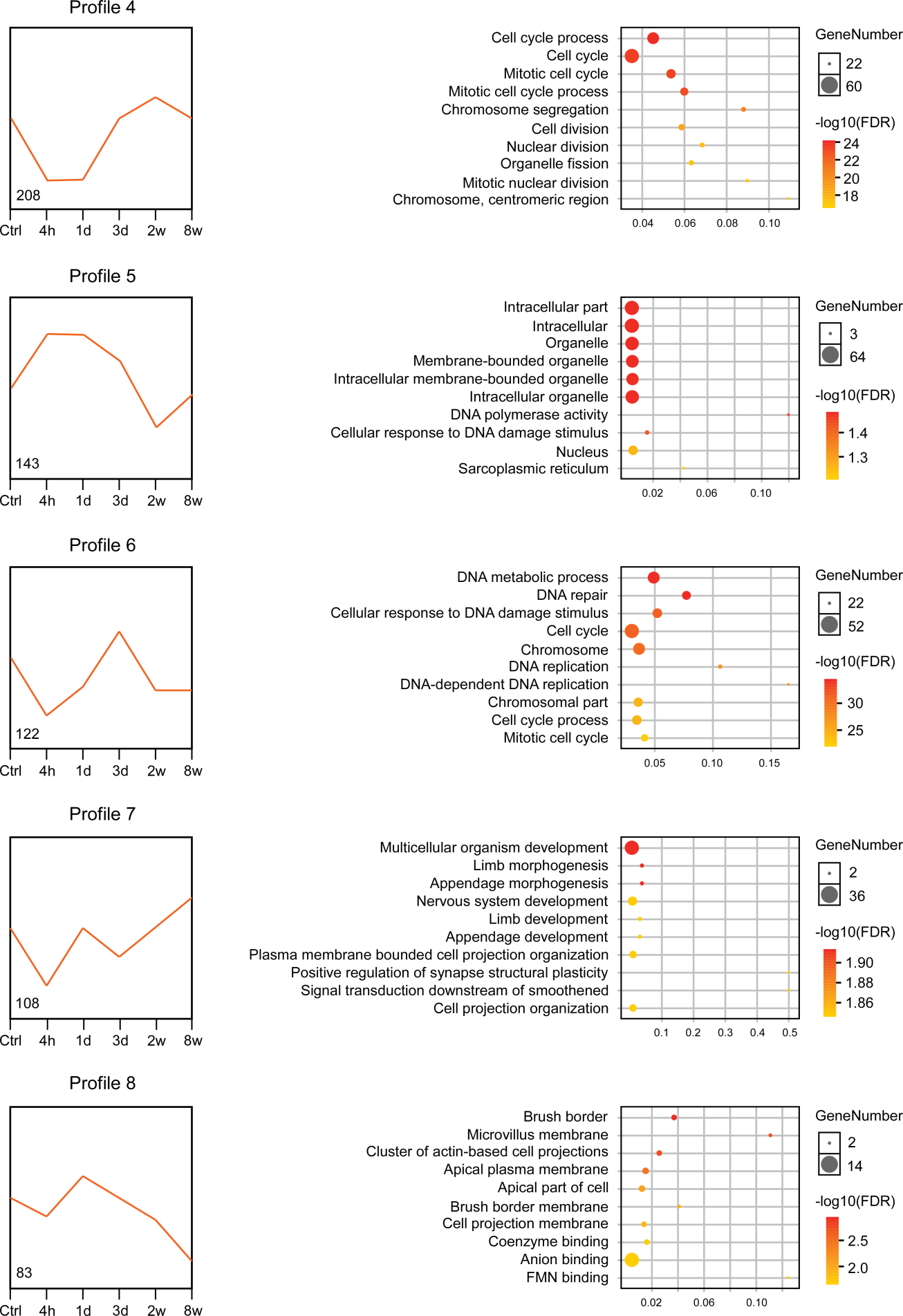
**

**Figure S1:** Gene profiles classified by STEM algorithm. The graphs (left) show the trends of gene expression change over time after exposure compared to sham-irradiated controls at 4 hours. For each profile, the vertical axis represents the gene expression levels based on FPKM and the horizontal axis represents time points post-irradiation. The number in the lower left of the box represents the gene number contained in each profile. The bubble chart (right) shows the top10 most enrichment GO terms in each profile.

**Supplementary Tables**

**Table S1.** The average values of topological features of the PPI networks for each time point.

| Time point | Average Shortest-Path Length | Betweenness Centrality | Closeness Centrality | Clustering Coefficient | Degree |
| --- | --- | --- | --- | --- | --- |
| 4 hours | 1.425 | 0.006 | 0.727 | 0.856 | 41.887 |
| 1 day | 1.800 | 0.100 | 0.577 | 0.352 | 3.400 |
| 3 days | 2.828 | 0.017 | 0.409 | 0.334 | 5.839 |
| 2 weeks | 2.281 | 0.014 | 0.460 | 0.540 | 13.093 |
| 8 weeks | 2.829 | 0.004 | 0.368 | 0.434 | 23.268 |

**Table S2.** Information for the human specimens.

| Specimen type | Gender | Age | Tumor type | Radiation dose/fractionation | Type of chemotherapy | Time tissue collected |
| --- | --- | --- | --- | --- | --- | --- |
| Normal control | Female | 68 | Rectal cancer | NA | NA | Immediately after resection of rectal cancer |
| ARP | Female | 70 | Rectal cancer | 50Gy/25F | FOLFOX | Two months after the last radiotherapy |
| CRP | Female | 59 | Cervical cancer | 54Gy/25F external and 30Gy/5F internal | Cis-platinum | One year after the last radiotherapy |

**Table S3.** Primer sequences for qRT-PCR.

| Gene name | Sequence (5`- 3`) | |
| --- | --- | --- |
| Actin-Mus | forward | GCAGATGTGGATCAGCAAGC |
|  | reverse | GCAGCTCAGTAACAGTCCGC |
| Cdk1-Mus | forward | AGGTACTTACGGTGTGGTGTAT |
|  | reverse | CTCGCTTTCAAGTCTGATCTTCT |
| Ccna2-Mus | forward | GCCTTCACCATTCATGTGGAT |
|  | reverse | TTGCTCCGGGTAAAGAGACAG |
| Aurkb-Mus | forward | CGGGAGAAGAAGAGCCGTTT |
|  | reverse | GTTGGGATGTTTCAGGTGCG |
| Aurka-Mus | forward | CTGGATGCTGCAAACGGATAG |
|  | reverse | CGCTGGGAGTTAGAAGGACAC |
| Mki67-Mus | forward | ATCATTGACCGCTCCTTTAGGT |
|  | reverse | GCTCGCCTTGATGGTTCCT |
| Mdm2-Mus | forward | TGTCTGTGTCTACCGAGGGTG |
|  | reverse | TCCAACGGACTTTAACAACTTCA |
| Cdkn1a-Mus | forward | CCTGGTGATGTCCGACCTG |
|  | reverse | CCATGAGCGCATCGCAATC |
| Bbc3-Mus | forward | CACCGAGATGTTGGCGTTTG |
|  | reverse | CCAGGCAAGCGACAGATACA |
| Bax-Mus | forward | AGACAGGGGCCTTTTTGCTAC |
|  | reverse | AATTCGCCGGAGACACTCG |
| Sesn2-Mus | forward | GAGTGCCATTCCGAGATCAAG |
|  | reverse | TAGTCCGGGTGTAGACCCATC |
| Tnf-Mus | forward | CCACGTCGTAGCAAACCACC |
|  | reverse | CCCTTGAAGAGAACCTGGGAG |
| Myc-Mus | forward | CCCTATTTCATCTGCGACGAG |
|  | reverse | GAGAAGGACGTAGCGACCG |
| Cd44-Mus | forward | AGAAAAATGGCCGCTACAGTATC |
|  | reverse | TGCATGTTTCAAAACCCTTGC |
| Col1a1-Mus | forward | TTCTCCTGGCAAAGACGGAC |
|  | reverse | CGGCCACCATCTTGAGACTT |
| Icam1-Mus | forward | TCCGCTACCATCACCGTGTAT |
|  | reverse | TAGCCAGCACCGTGAATGTG |
| Fn1-Mus | forward | ATGTGGACCCCTCCTGATAGT |
|  | reverse | GCCCAGTGATTTCAGCAAAGG |
| Cdh5-Mus | forward | CCAACGTGAACCGCCAGAA |
|  | reverse | GTGTTAGCATCGACCCCGAA |
| Pecam1-Mus | forward | ACCGGGTGCTGTTCTATAAGG |
|  | reverse | TCACCTCGTACTCAATCGTGG |
| Mmp2-Mus | forward | CAAGTTCCCCGGCGATGTC |
|  | reverse | TTCTGGTCAAGGTCACCTGTC |
| Alb-Mus | forward | CAAGAGTGAGATCGCCCATCG |
|  | reverse | TTACTTCCTGCACTAATTTGGCA |
| Il6-Mus | forward | CCCCAATTTCCAATGCTCTCC |
|  | reverse | CGCACTAGGTTTGCCGAGTA |
| Il1b-Mus | forward | TTCAGGCAGGCAGTATCACTC |
|  | reverse | GAAGGTCCACGGGAAAGACAC |
